# Novel glassbox based explainable boosting machine for fault detection in electrical power transmission system

**DOI:** 10.1371/journal.pone.0309459

**Published:** 2024-08-28

**Authors:** Iqra Akhtar, Shahid Atiq, Muhammad Umair Shahid, Ali Raza, Nagwan Abdel Samee, Maali Alabdulhafith

**Affiliations:** 1 Department of Electrical and Biomedical Engineering, Khwaja Fareed University of Engineering and Information Technology, Rahim Yar Khan, Pakistan; 2 Department of Software Engineering, University Of Lahore, Lahore, Pakistan; 3 Department of Information Technology, College of Computer and Information Sciences, Princess Nourah bint Abdulrahman University, Riyadh, Saudi Arabia; Graphic Era Deemed to be University, INDIA

## Abstract

The reliable operation of electrical power transmission systems is crucial for ensuring consumer’s stable and uninterrupted electricity supply. Faults in electrical power transmission systems can lead to significant disruptions, economic losses, and potential safety hazards. A protective approach is essential for transmission lines to guard against faults caused by natural disturbances, short circuits, and open circuit issues. This study employs an advanced artificial neural network methodology for fault detection and classification, specifically distinguishing between single-phase fault and fault between all three phases and three-phase symmetrical fault. For fault data creation and analysis, we utilized a collection of line currents and voltages for different fault conditions, modelled in the MATLAB environment. Different fault scenarios with varied parameters are simulated to assess the applied method’s detection ability. We analyzed the signal data time series analysis based on phase line current and phase line voltage. We employed SMOTE-based data oversampling to balance the dataset. Subsequently, we developed four advanced machine-learning models and one deep-learning model using signal data from line currents and voltage faults. We have proposed an optimized novel glassbox Explainable Boosting (EB) approach for fault detection. The proposed EB method incorporates the strengths of boosting and interpretable tree models. Simulation results affirm the high-efficiency scores of 99% in detecting and categorizing faults on transmission lines compared to traditional fault detection state-of-the-art methods. We conducted hyperparameter optimization and k-fold validations to enhance fault detection performance and validate our approach. We evaluated the computational complexity of fault detection models and augmented it with eXplainable Artificial Intelligence (XAI) analysis to illuminate the decision-making process of the proposed model for fault detection. Our proposed research presents a scalable and adaptable method for advancing smart grid technology, paving the way for more secure and efficient electrical power transmission systems.

## Introduction

In the era of Industry 4.0, there is a continuous increase in the demand for electrical power. To accommodate the growing need for energy, there is an increase in power generation facilities. These facilities are linked together via an intricate network known as the power system network (PSN), which is composed of three primary elements: generation of power, its transmission, and distribution [1]. Power is transported between locations via transmission lines. The utilization of high-capacity electrical power plants and the concept of a synchronized grid involving geographically dispersed power sources [2] necessitates the rapid detection of faults and the operation of protective equipment to maintain a stable power system. The protection system employed for transmission lines can also trigger other relays to safeguard the power system against disruptions [3]. It is essential to maintain a stable and dependable power system to mitigate its effects on sectors such as industries, commerce, transportation, and residential areas. Thus, ensuring uninterrupted and secure power transmission is of utmost importance for a country’s economic activities.

**Fig 1:**
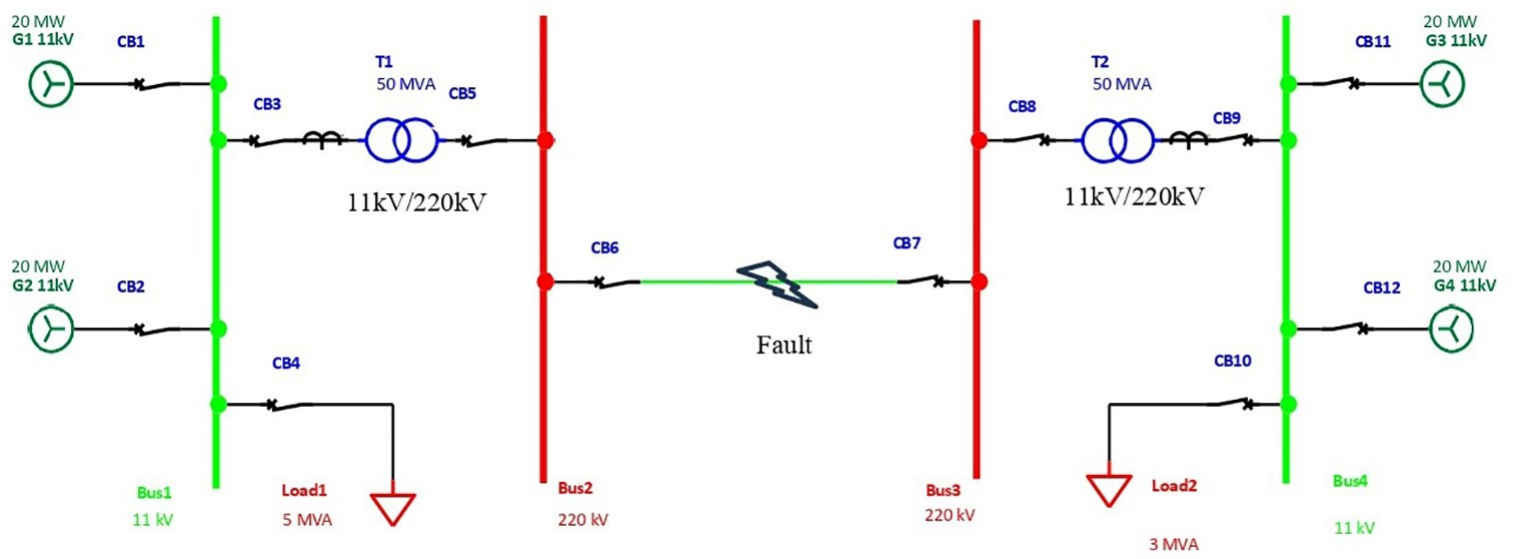
The single line workflow analysis in electrical transmission.

The capability of transmission lines to transfer power can often be compromised by open and short-circuit faults [4]. These faults in the power system can arise from natural events such as lightning, strong winds, and earthquakes or accidents like trees falling, vehicle collisions, and plane crashes [5]. Natural events can lead to short circuits, which may manifest as phase-to-phase faults, single-phase-to-ground faults, phase-to-phase-to-ground faults, or three-phase faults [6]. In electrical systems with overhead lines, single-phase-to-ground faults are most commonly triggered by transient high voltages induced by lightning and tree contact due to wind, as shown in Fig 1. Additionally, the early stages of faults can be initiated by partial discharge (PD) activities in power system components.

The repercussions of faults include a significant increase in current flow, resulting in elevated heat generation in the conductors, which is a primary cause of damage [7]. The actual fault magnitude depends on the resistance to current flow and the varied impedance between the fault and the power supply source [8]. This total impedance encompasses fault resistance, conductor line resistance and reactance, transformer impedance, circuit reactance, and generating station impedance. Conventional distance relay settings are traditionally established based on a predetermined network configuration, considering the worst possible fault scenarios.

A short-circuit breakdown causes a massive loss of power [9]. Accurate identification of the fault type and its exact position is crucial for maintaining stability. Any malfunction in the electrical system can lead to severe disruptions and financial losses. When faults arise within the power system, notable variations in system parameters like current overload, power factor, power imbalance, frequency, impedance, and the direction of power or current commonly occur.

Utilizing pattern recognition methods can prove advantageous for identifying abnormalities as well as normal conditions within electrical power systems [10]. Furthermore, this approach facilitates the identification of the specific phase in a three-phase electrical system that is under distress. The application of Artificial Neural Networks (ANNs) has shown remarkable efficiency in detecting anomalies and categorizing faults through pattern recognition techniques [4]. Many ANN-based algorithms have been created, tested, and effectively used in electrical power systems [11]. Within the present study, a new ANN-based technique for quick and reliable defect recognition and categorization is proposed. It is beneficial as it is a computing methodology that can readily tackle nonlinear problems. The challenges that can be addressed when there are a lot of details present. The numerous electrical transient system defects are represented and simulated, and an ANN-based approach for detecting these defective sequences is devised. The suggested algorithm’s efficacy is examined by recreating various kinds of errors, and the findings are favourable.

The primary contributions of our research study are followed as:

- The line currents and voltage fault data modeled in MATLAB for efficient electrical fault analysis within a power system. We analyzed the Signal Data Time Series Analysis based on Phase line current and Phase line voltage. We have balanced the dataset using SMOTE-based data oversampling.
- A novel Optimized Glassbox-based Explainable Boosting approach is proposed for fault detection in electrical power transmission systems. The proposed EB method combines the strengths of boosting and interpretable tree models. Additionally, we applied Explainable Artificial Intelligence (XAI) analysis to provide insights into how the proposed model makes decisions for fault detection.
- We have employed four recent machine learning models and a deep learning model. The hyperparameter optimization and k-fold validations are performed for fault detection performance enhancement and validation. We also determined the computational complexity of applied models for fault detection.

The remaining research body is assembled as follows: Section “Literature Analysis” contains the electrical faults-related work analysis. Section “Proposed Methodology” is based on the study methods for fault detection. Section “Results and discussions” provides the results validations and detailed discussion. The study is summed up in Section “Conclusions”.

## Literature analysis

The analysis of related work in research on fault detection in electrical power transmission systems with machine learning is critical for establishing the contextual framework and understanding the existing efforts in the field. A considerable amount of research has been conducted on the utilization of machine learning methods to identify faults within power systems, emphasizing the need for robust and efficient methodologies to enhance the reliability of electrical grids. Some studies have concentrated on feature engineering and selection approaches to optimize the performance results of these algorithms in detecting faults, as shown in Table 1.

**Table 1:** The state of art research studies analysis for fault detection in electrical power transmission systems.

Ref.	Year	DataSet Used	Proposed Technique	Performance Accuracy
[11]	2015	Three phase voltages and three phase currents	ANN	78%
[12]	2018	Electrical system inputs	ANN	80%
[13]	2018	Current, Voltage and active power	LSTM network and SVM	97%
[14]	2019	Incomplete and noisy data	ANN	84%
[15]	2022	Data of different SNRs	TSVD-HUARPANN Technique	98%
[16]	2022	Frequency response curve	CNN-LSTM	98%

In this [11] study, ANNs were employed for detecting and categorizing faults in a three-phase transmission line system, thereby enhancing power system reliability. The method relies on the normalization of a collection of currents and voltages as inputs, derived from their pre-fault values. Different fault types, such as double-line-to-ground, line-to-line, and symmetrical three-phase faults, are individually analyzed using dedicated ANNs. Simulations are conducted in MATLAB’s Simulink with the Sim Power Systems Toolbox. The proposed approach demonstrates optimistic results with a 78% accuracy score, showcasing the robust generalization capability of ANNs. Achieving notable accuracy, the ANN models exhibit effectiveness even with a limited 20% training data volume. The method is proven to accurately detect and classify various faults, including random vectors introduced anywhere in the system. In summary, the developed ANN model successfully localizes and classifies faults in a three-phase transmission line system.

In the study [12], the researchers explored the utilization of neural network applications specifically for fault diagnosis and detection, focusing on their implementation in engineering-related systems. Utilizing a dataset derived from electrical systems, the study aimed to construct an ANN methodology tailored for efficient fault diagnosis. The authors proposed an ANN framework designed to enhance fault classification accuracy. The findings of the research indicated that the ANN approach achieved a performance score of approximately 80%. However, this level of accuracy is considered relatively low, and it is associated with high error rates, suggesting a need for further optimization of the neural network model to improve its diagnostic capabilities in engineering applications.

In the study [15], a machine learning-based hybrid approach aimed at detecting transmission line faults in power systems. The research utilized data across different Signal-to-Noise Ratios (SNRs) to carry out the experiments. They proposed a novel hybrid technique, termed TSVD-HUARPANN, for fault detection. The experimental results of this study demonstrated that the proposed approach achieved an accuracy score of 98%, indicating a moderate level of precision in fault detection. However, there is potential for further enhancing performance by incorporating more advanced techniques, suggesting that while the results are promising, there is still scope for improvement in the methodology.

In the study [14], an innovative method for fault localization and classification in power transmission lines was introduced, leveraging an intelligent approach. The research capitalized on analyzing incomplete and noisy data drawn from a vast array of transmission and distribution lines. The use of an Artificial Neural Network (ANN) has been proposed as a method to accurately classify and pinpoint faults within the power transmission infrastructure. Achieving an 84% accuracy rate in fault detection through their method. However, this level of performance, while notable, suggests there is significant room for improvement, particularly in enhancing the detection capabilities for critical fault identification in such systems. This indicates a potential avenue for further research aimed at refining the fault detection process to ensure more reliable and efficient power transmission systems.

In the study [13], use of machine learning-based approaches to predict line trip faults in power systems. Implemented deep learning-based Long Short-Term Memory (LSTM) networks along with Support Vector Machine (SVM) models for efficient fault detection. The research comprised datasets comprising current, voltage, and active power measurements to develop and validate the proposed methods. The results scores of this research indicated that the combined use of LSTM networks and SVM models achieved a remarkable fault detection accuracy rate of 97%. Despite the high performance, there remains potential for further enhancements in detection accuracy, suggesting that the field could benefit from continued research and development to optimize these predictive models.

### Research gaps and limitations

We have identified the following research gaps through the above literature analysis:

- In current literature, classical ANN-based approaches were built for the detection of electrical faults in transmission systems such as linear SVM and tree-based models.
- There is still a need for performance enhancement for critical electrical fault detection in transmission systems. Moderate performance accuracy of 98% in literature is achieved, however, the 2% error still exists, which can be further overcome by advanced machine learning models.
- Current and voltages signals data patterns were not identified to apply appropriate machine learning approaches,

## Proposed methodology

**Fig 2:**
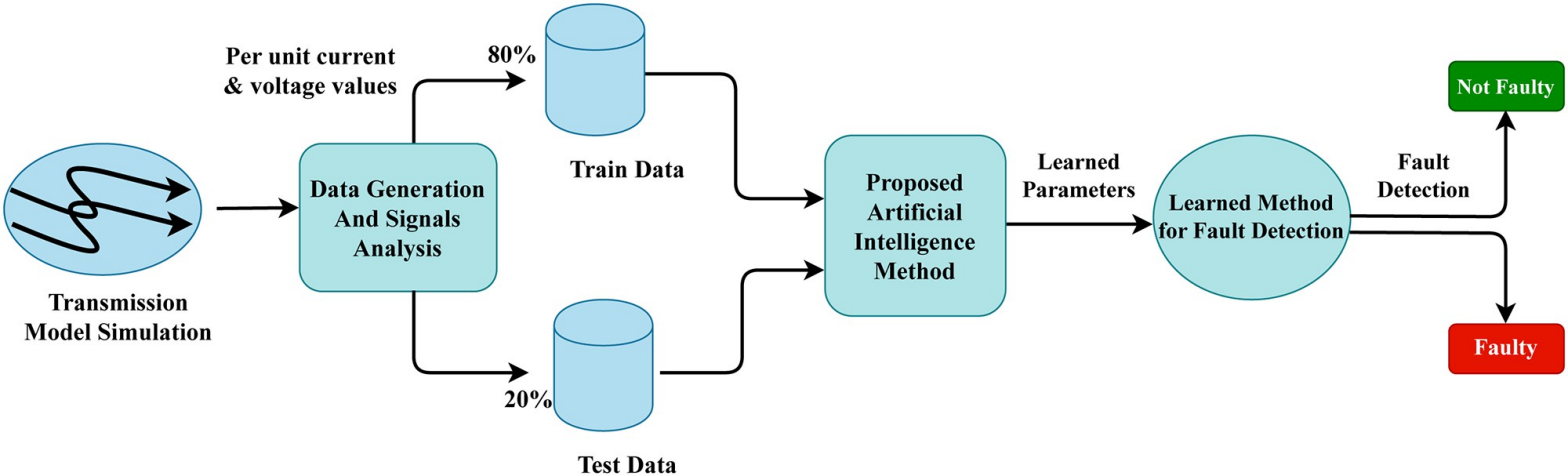
The proposed electrical fault detection abstract workflow analysis.

In this section, the materials and methods are meticulously outlined to detail the comprehensive approach undertaken for detecting electrical faults in power transmission systems. This involved utilizing line currents and voltage fault data, which is carefully curated to ensure it represented a comprehensive sample of the population under study, as shown in abstract Fig 2.

**Fig 3:**
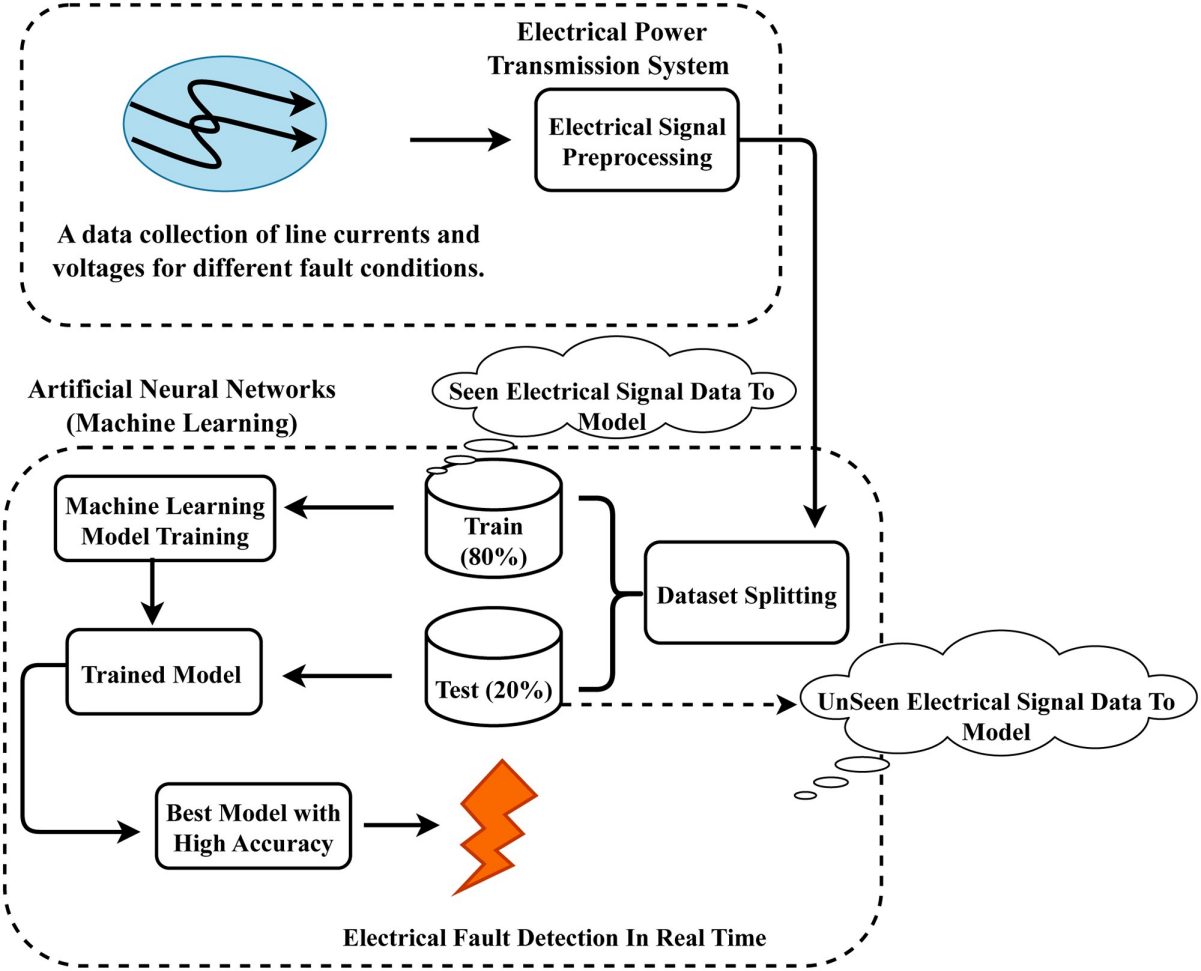
The proposed electrical fault detection stepwise workflow analysis.

Fig 3 illustrates our proposed innovative workflow architecture for electrical fault detection in power systems. Initially, data based on line currents and voltage signal values from transmission model simulations are utilized to commence research experiments. The line currents and voltage fault signal data is analyzed using a time-series signal space analysis, which infers the patterns during electrical faults. Then, the formatted dataset is split into train (80%) and test (20%) portions. We have trained several advanced artificial intelligence methods and evaluated them using the testing dataset. We have tuned the hyperparameters of the applied methods to enhance electrical fault detection. The best-performing artificial intelligence approach is then utilized in real-time for the detection of electrical faults in a power transmission system.

### Phase 1: Line currents and voltages faults data

This research utilized a benchmark dataset [11, 17], comprising a collection of line currents and voltages under various fault conditions in the power transmission system. The data were modelled in MATLAB to simulate fault analysis within a power system framework. The modelled power system incorporates four generators, each with a voltage of 11 × 10^3^ V, positioned in pairs at both ends of the line transmission. The dataset is based on 12,001 transmission line data points. Transformers are also included in the power system to facilitate the simulation and analysis of numerous faults occurring at the middle of the line transmission.

The dataset was collected and recorded, detailing the measured line currents and line voltages at the output side of the power system. This collection encompasses nearly 12,000 data points, subsequently labelled as either ‘fault’ or ‘no fault’. The simulation inputs are designated as Phase A line current (Ia), Phase B line current (Ib), Phase C line current (Ic), Phase A line voltage (Va), Phase B line voltage (Vb), and Phase C line voltage (Vc). The outputs are categorized as 0 (No fault) or 1 (Fault present) in the power transmission system, as shown in Table 2.

**Table 2:** Different types of faults analysis in the three-phase transmission line in the dataset.

Fault Name	Value
No Fault	[0 0 0 0]
LG Fault (In Between Phase A and Ground)	[1 0 0 1]
LL Fault (In Between Phase A and B)	[0 0 1 1]
LLG Fault (In Between Phase A, B and Ground)	[1 0 1 1]
LLL Fault (In Between All Three Phases)	[0 1 1 1]
LLLG Fault (In Three Phase Symmetrical Fault)	[1 1 1 1]

### Phase 2: Signal data time series analysis

**Fig 4:**
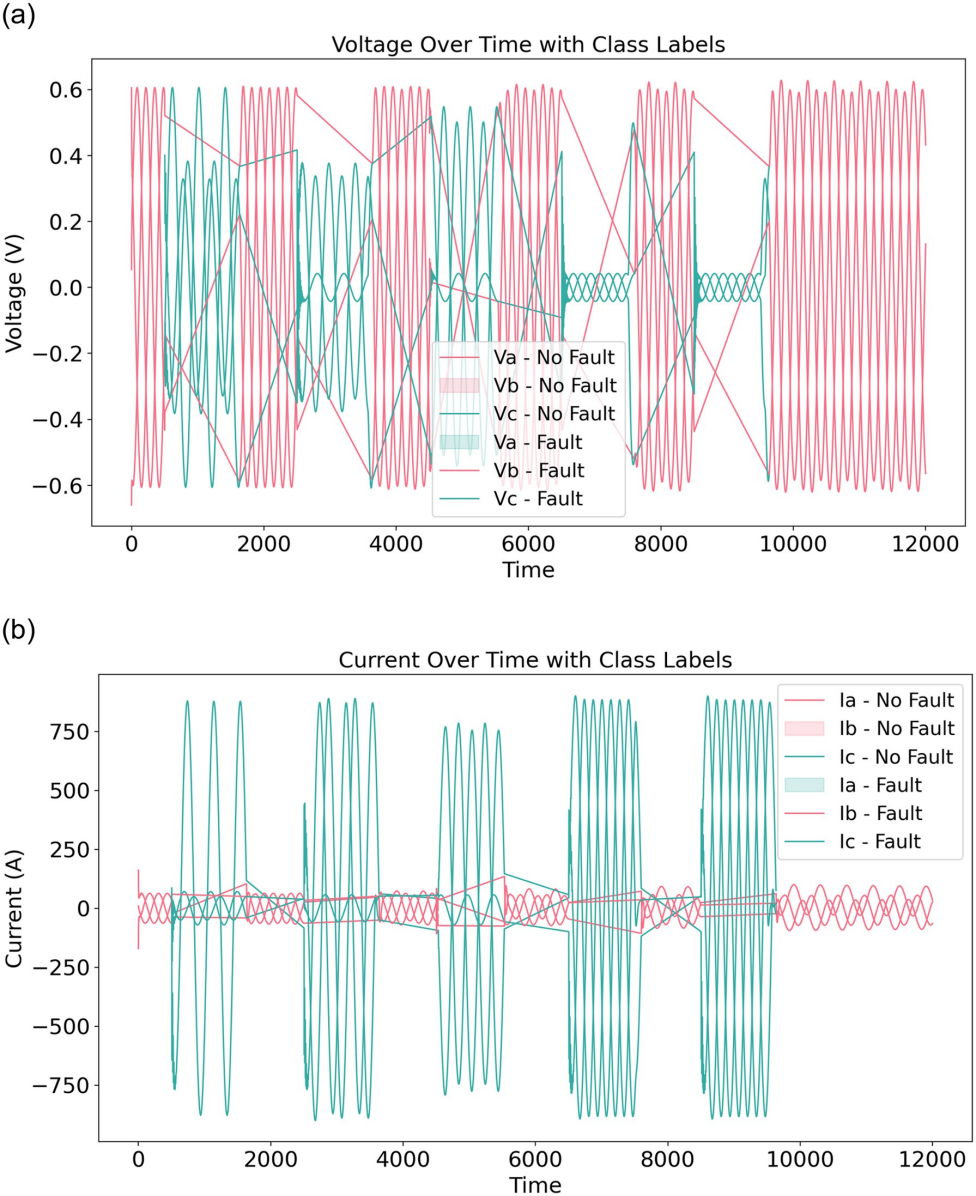
The voltage and current analysis overtime during a fault or not fault in electrical power transmission systems.

We have analyzed the signal data for faults over the transmission line. The analysis of voltage and current over time, during a fault or no-fault in electrical power transmission systems, is shown in Fig 4. The voltage signals over time are depicted in Fig 4(a). This analysis indicates that when Va voltage values are in the range of -0.6 to 0.6, there is no fault. However, when the Va is near 0.0, it means there is a fault over the transmission line. Similarly, this applies to Vb and Vc. The current signals over time are depicted in Fig 4(b). The analysis also shows that when Ia current values are near 0.0, there is no fault. However, when the Ia is in the range of -750 to 750, it means there is a fault over the line transmission. A similar pattern is observed for Ib and Ic.

### Phase 3: SMOTE based data oversampling

**Fig 5:**
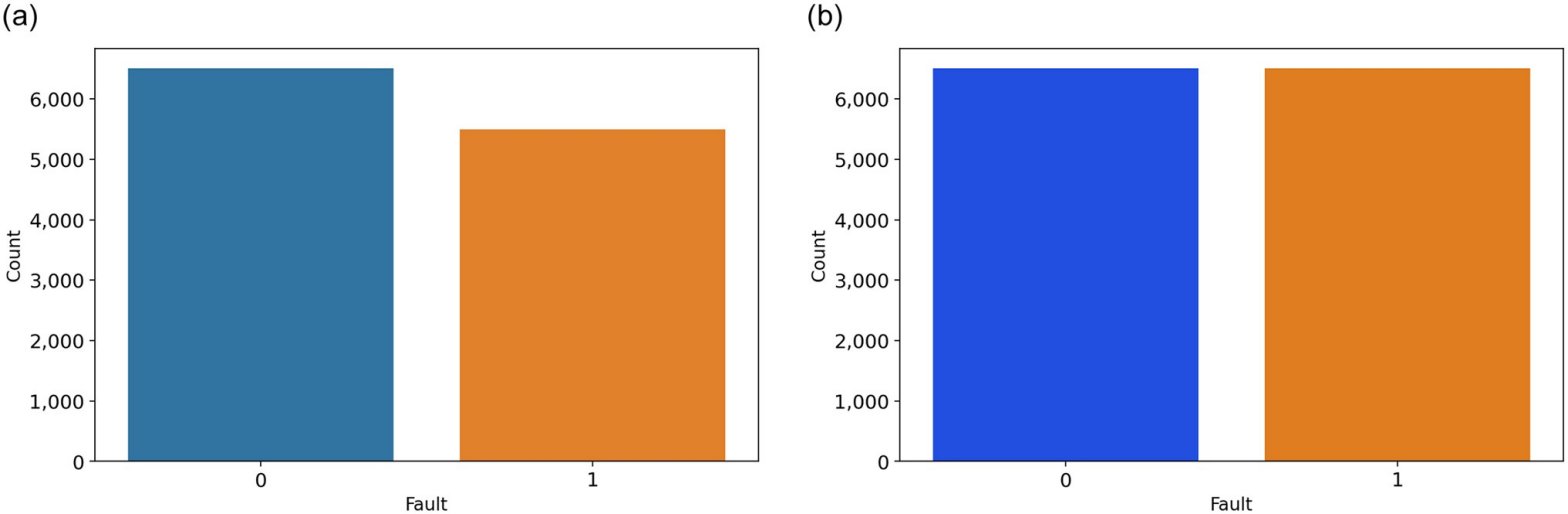
The fault class distribution analysis before and after balancing.

After analyzing the signal data, we have determined that the dataset is unbalanced, as illustrated in Figure Fig 5(a). Then, we employed a well-known dataset oversampling technique, the Synthetic Minority Over-sampling Technique (SMOTE) [18]. The SMOTE approach, which focuses on data oversampling of line currents and voltage faults, emerges as a pivotal method for enhancing fault detection accuracy in electrical power transmission systems. By addressing the imbalance in datasets, where instances of faults are significantly underrepresented compared to normal operation data, SMOTE facilitates the generation of synthetic samples. The balanced dataset, after applying the SMOTE approach, is illustrated in Fig 5(b).

### Phase 4: Signal data splitting

The signal data is then split to conduct further research experiments. We used a splitting ratio of 80:20. The dataset is divided into train (80%) and test (20%) portions. This research process is fundamental in avoiding overfitting, ensuring that the model can generalize well to new, unseen data by training the model on one subset of the data while validating and testing its performance on other subsets.

### Phase 5: Applied artificial intelligence methods

Machine learning methods play a pivotal role in enhancing fault detection capabilities within electrical power transmission systems. By leveraging advanced algorithms [19–21], these systems can analyze vast datasets in real time, identifying subtle deviations from normal operating conditions. This proactive approach enables swift and accurate detection of potential faults, minimizing downtime and ensuring the reliability of power transmission networks.

- **Random Forest (RF):** The RF method for Fault Detection in Electrical Power Transmission Systems operates by leveraging a combination of decision trees [22, 23]. Each tree is created using a random subset of features and a bootstrapped sample of the dataset, ensuring diversity in the individual models. During fault detection, the RF algorithm aggregates predictions from multiple trees, providing a robust and accurate assessment of the system’s health. This ensemble approach enhances the model’s resilience to noise and variability, making it particularly effective for detecting faults in complex and dynamic electrical transmission environments. Mathematically, the prediction *y* for a given input vector *X* can be represented as:
  $$\begin{array}{c} y=\frac{1}{N}\sum_{i=1}^{N}T_{i}\left(X;\Theta_{i}\right) \end{array}$$ (1)
  Where:
  - *N* is the number point of trees in the forest.
  - *T*_*i*_(*X*;Θ_*i*_) is the prediction of the *i*^*th*^ tree, trained with a randomly sampled dataset (with replacement) from the training set and feature subset, based on parameters Θ_*i*_.
  - *X* represents the input features vector derived from the power system’s parameters.
  For fault detection, the output class can be determined based on majority voting among the trees:
  $$\begin{array}{c} \text{Class}\left(X\right)=\text{mode}\{T_{1}\left(X\right),T_{2}\left(X\right),\ldots ,T_{N}\left(X\right)\} \end{array}$$ (2)
  Where Class(*X*) represents the predicted state (e.g., ‘normal’ or ‘faulty’) of the electrical power transmission system.
- **Logistic Regression (LR):** The LR operates as a powerful approach for Fault Detection in Electrical Power Transmission Systems by employing a probabilistic approach [24, 25]. It assesses the likelihood of a fault occurrence based on input features, such as voltage levels and current values. The LR model employs a logistic function to map the input data into a probability distribution, enabling the identification of potential faults through a threshold classification. This method proves effective in discerning abnormal patterns within the system, facilitating timely intervention and maintenance in power transmission networks. The logistic function, *σ*(*z*), is defined as:
  $$\begin{array}{c} \sigma \left(z\right)=\frac{1}{1+e^{-z}} \end{array}$$ (3)
  where *z* data point is the linear data combination value of the input features data (*x*_1_, *x*_2_, …, *x*_*n*_) and their corresponding weights (*w*_1_, *w*_2_, …, *w*_*n*_), plus a bias term *b*. This can be expressed as:
  $$\begin{array}{c} z=w_{1}x_{1}+w_{2}x_{2}+\ldots +w_{n}x_{n}+b \end{array}$$ (4)
  Substituting *z* in the logistic function:
  $$\begin{array}{c} \sigma \left(z\right)=\frac{1}{1+\text{exp}(-(w_{1}x_{1}+w_{2}x_{2}+\ldots +w_{n}x_{n}+b))} \end{array}$$ (5)
  This model outputs the probability that a given set of input features (*x*_1_, *x*_2_, …, *x*_*n*_) belongs to the “fault” category. To classify an input as “fault” or “no fault”, a threshold, typically 0.5, is applied to the model’s output:
  $$\begin{array}{c} \text{Prediction}=\{\begin{array}{cc} “\text{fault}” & \text{if}\;\sigma (z)\geq 0.5\\ “\text{no}\;\text{fault}” & \text{if}\;\sigma (z)<0.5 \end{array} \end{array}$$ (6)
- **Support Vector Classifier (SVC):** The SVC operates by constructing a hyperplane in a space that effectively separates different classes of data points [26, 27]. In the context of fault detection in electrical power transmission systems, the SVC method utilizes historical data on normal system behavior and fault instances to define this hyperplane. By identifying the optimal hyperplane that maximally separates normal and faulty conditions, SVC enables efficient detection of abnormalities, enabling swift response to potential faults in the power transmission system. This method’s robustness lies in its ability to handle non-linear relationships and its sensitivity to critical features, enhancing its efficacy in safeguarding the electrical grid against potential faults. The fault detection in electrical power transmission systems, let’s consider the feature space to be $x\in R^{n}$ and the two categories to be *fault* and *no fault*.
  The basic form of the SVC decision function is:
  $$\begin{array}{c} f\left(x\right)=w^{⊤}x+b \end{array}$$ (7)
  where:
  - $w\in R^{n}$ is the weight data vector perpendicular to the SVC hyperplane.
  - b∈R is the bias term, which offsets the hyperplane from the origin.
  - $x\in R^{n}$ is the input feature vector.
  The goal of the SVC is to find the optimal data points **w** and *b* such that the margin between the two classes is maximized, subject to the constraints:
  $$\begin{array}{c} y_{i}\left(w^{⊤}x_{i}+b\right)\geq 1,\;\forall i \end{array}$$ (8)
  where *y*_*i*_ ∈ {−1, 1} is the label of **x**_*i*_, indicating the class (fault or no fault).
  The optimization problem for finding **w** and *b* can be formulated as:
  $$\begin{array}{c} \underset{w,b}{\text{min}}\frac{1}{2}{∥w∥}^{2} \end{array}$$ (9)
  subject to:
  $$\begin{array}{c} y_{i}\left(w^{⊤}x_{i}+b\right)-1\geq 0,\;\forall i \end{array}$$ (10)
  This formulation ensures that the hyperplane has the maximum line margin separating the two classes, which is a key concept in SVC for fault detection and classification tasks.
- **Gated Recurrent Unit (GRU):** The GRU deep learning model emerges as a potent tool for fault detection in electrical transmission systems [28, 29]. Leveraging its ability to capture temporal dependencies in data, the GRU efficiently processes sequential information from power transmission measurements, enabling the detection of subtle deviations indicative of faults. This model’s adaptability and streamlined architecture make it a promising solution for enhancing the reliability and efficiency of fault detection in critical electrical infrastructure. The fundamental equations governing the GRU operation are as follows:
  $$\begin{array}{c} r_{t}=\sigma (W_{r}\cdot \left[h_{t-1},x_{t}\right]+b_{r}) \end{array}$$ (11)
  $$\begin{array}{c} z_{t}=\sigma (W_{z}\cdot \left[h_{t-1},x_{t}\right]+b_{z}) \end{array}$$ (12)
  $$\begin{array}{c} {\tilde{h}}_{t}=\text{tanh}(W\cdot \left[r_{t}⊙h_{t-1},x_{t}\right]+b) \end{array}$$ (13)
  $$\begin{array}{c} h_{t}=\left(1-z_{t}\right)⊙h_{t-1}+z_{t}⊙{\tilde{h}}_{t} \end{array}$$ (14)
  Where:
  - *r*_*t*_ is the data value of the reset gate activation vector.
  - *z*_*t*_ is the data value of the update gate activation vector.
  - *h*_*t*_ is the data value of the hidden state vector at time *t*.
  - *x*_*t*_ is the input data vector at time *t*.
  - ${\tilde{h}}_{t}$ is the candidate activation vector.
  - *σ* denotes the sigmoid value function.
  - ⊙ denotes data element-wise multiplication.
  - *W*_*r*_, *W*_*z*_, *W* and *b*_*r*_, *b*_*z*_, *b* are the weights and biases that need to be learned during training.

The GRU’s ability to effectively blend past information with current inputs through its gating mechanisms makes it particularly suited for detecting anomalies over time in power transmission systems. The layered architecture is analyzed in Table 3.

**Table 3:** The layers architecture stack of applied neural network based GRU method.

Layer Name	Output Shape	Param#
GRU layer	(None, 4)	84
Dense layer	(None, 32)	160
Output Dense layer	(None, 1)	33
Total Params	277	

### Phase 6: Novel glassbox-based proposed EB approach

The proposed EB approach for fault detection in electrical power transmission systems employs an ensemble of decision trees to analyze the system’s data. The proposed EB method combines the strengths of boosting and interpretable models as glassbox, providing a transparent and accurate framework for fault detection. Through iterative training, the EB model assigns weights to individual decision trees, emphasizing the contribution of each tree to the overall predictive outcome, facilitating a clear understanding of the features and patterns crucial for detecting faults in the power transmission system. The basic form of a model under the EB approach, adapted for fault detection, can be represented as follows:

$$\begin{array}{c} \hat{y}=\varphi_{0}+\sum_{i=1}^{M}\varphi_{i}\left(x_{i}\right)+\sum_{i,j=1i<j}^{M}\varphi_{ij}\left(x_{i},x_{j}\right)+\epsilon \end{array}$$ (15)

Where:

-
$\hat{y}$ is the predicted outcome (e.g., the presence or absence of fault data in the power transmission system).
- *φ*_0_ is the model’s intercept.
- *M* is the number of features (e.g., voltage, current, temperature, etc.) used in the model.
- *x*_*i*_ represents an individual feature.
- *φ*_*i*_(*x*_*i*_) is the contribution values of the *i*-th feature to the prediction, modeled by a shape function, which is learned during training for maximum interpretability.
- *φ*_*ij*_(*x*_*i*_, *x*_*j*_) represents the interaction term between features *i* and *j*, capturing the joint effect of these features on the prediction. This term allows the model to consider how combinations of different conditions affect the system’s health.
- *ϵ* is the error term.

**Fig 6:**
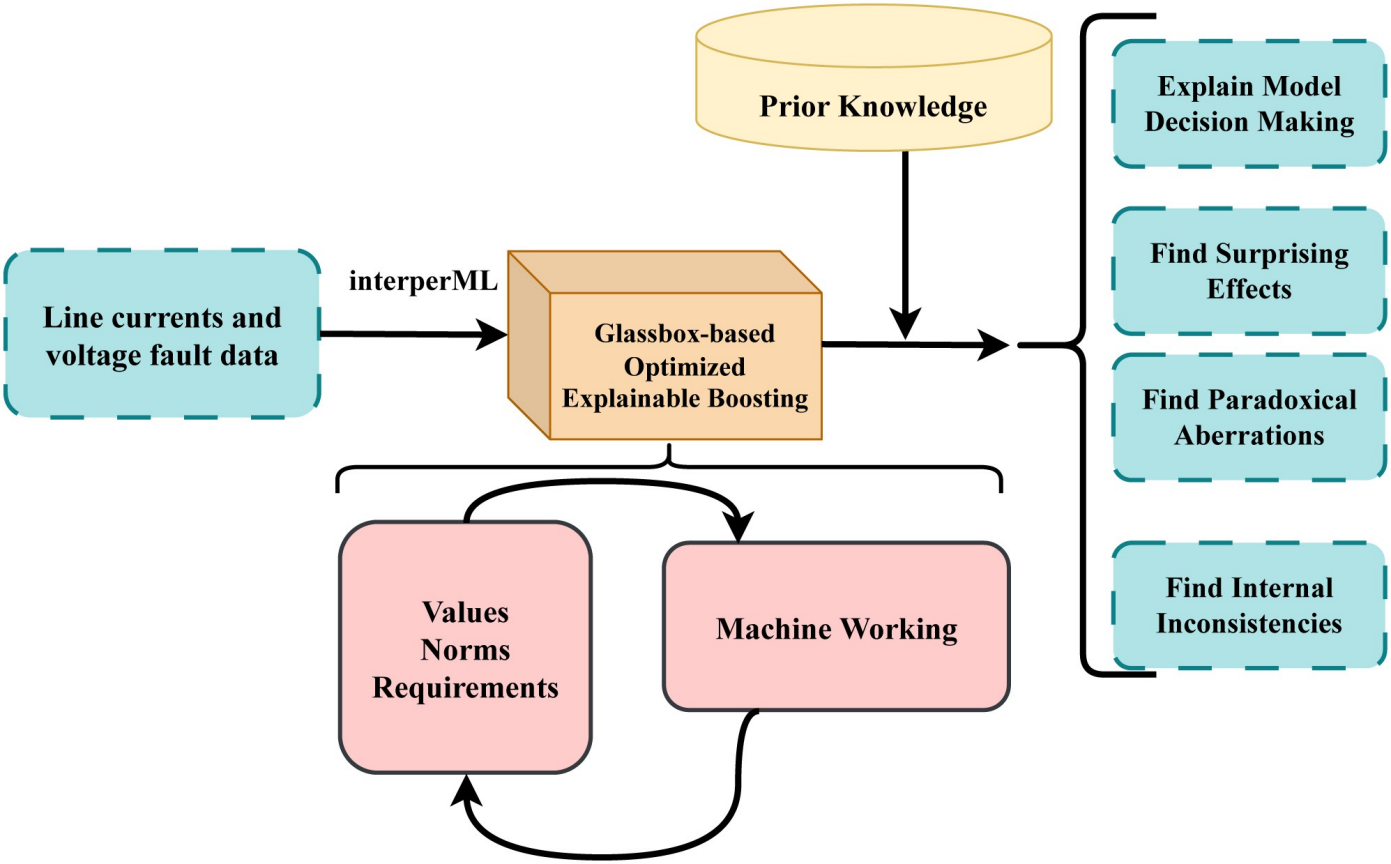
The proposed glassbox-based optimized explainable boosting workflow analysis.

Our proposed methodology enables the development of boosting models that are not only highly accurate in identifying and classifying electrical faults but also provide clear insights into their decision-making processes. By leveraging the glassbox model, we can pinpoint the specific characteristics and conditions under which faults occur, facilitating more effective prevention strategies. This approach significantly enhances the reliability and safety of electrical power transmission systems, offering a clear window into the inner workings of complex AI-driven fault detection mechanisms. The architecture of the proposed approach is illustrated in Fig 6 and Algorithm 1.

**Algorithm 1** Training proposed EB Classifier for Fault Detection in Power Transmission Systems

**Input:** Dataset *D* containing line currents and voltages under various fault conditions. Each instance *x*_*i*_ has a label *y*_*i*_ where *y*_*i*_ ∈ {0, 1} (0 for No fault, 1 for Fault present).

**Output:** A trained Explainable Boosting Classifier model

Initialize the Explainable Boosting Classifier *M*

Configure model parameters (if any specific configuration is needed)

Train the model *M* using the dataset *D*

Evaluate the model performance using suitable metrics (e.g., accuracy, F1-score)

**return** *Trained Model M*

### Phase 7: Models perparameters settings

This section analyzes the hyperparameter tuning of applied machine-learning models for electrical fault detection. Hyperparameter tuning helps to improve the generalization of applied machine learning models and prevents overfitting issues. The best-fit hyperparameters in this study are determined through a recursive training and testing process using the k-fold cross-validation process. The final selected hyperparameter settings of the applied method are described in Table 4.

**Table 4:** Analysis of parameter optimization in machine learning applications.

Technique	Hyperparameters Description
RF	n_estimators = 4, max_depth = 3, random state = 0, criterion=‘entropy’
LR	random_state = 0, solver=‘lbfgs’, max_iter = 500, multi_class=‘auto’, C = 1.0
SVC	kernel = linear, Random_state = 0, penalty=‘l2’, loss=‘squared_hinge’, dual=‘warn’,max_iter = 300
GRU	loss = binary_crossentropy, activation = sigmoid, optimizer = adam, epochs = 5, Trainable params: 277 (1.08 KB)
EB	max_bins = 256, max_interaction_bins = 32, interactions = 10, learning_rate = 0.01, max_rounds = 5000, min_samples_leaf = 2, max_leaves = 3, objective=‘log_loss’, n_jobs=-2,

## Results and discussions

In this section, neural network methods for fault detection in electrical power transmission systems yielded promising outcomes. The implemented algorithms demonstrated high accuracy in identifying and classifying faults, showcasing their potential for enhancing the reliability of the system. The discussion delves into the intricacies of model performance, highlighting areas of improvement and offering insights into the practical implications of integrating machine learning into fault detection strategies for power transmission systems.

### Experimental setup

This section scrutinizes the experimental setup of our study. We utilized Python programming language 3.0 to implement the applied machine learning approaches. All experiments are conducted in the Google Colab platform, utilizing a GPU backend with 90 GB disk space and 13 GB RAM. Established statistical metrics like accuracy, F1, precision, and recall are employed to evaluate the models. The basic evaluation terms are based on False Positives (FP), True Positives (TP), True Negatives (TN), and False Negatives (FN) for each metric. The mathematical representation of these evaluation criteria, such as accuracy, is provided:

$$\begin{array}{c} Accuracy=\frac{TP+TN}{TP+TN+FP+FN} \end{array}$$ (16)

Where TP denotes correctly identified faults, TN denotes correctly identified non-faults, FP denotes incorrectly identified faults, and FN denotes incorrectly missed faults, respectively.

Similarly, precision is the ratio of TP to the sum of TP and FP, denoted as precision (P). It represents the ratio of accurately predicted erroneous data to the total data predicted to be erroneous:

$$\begin{array}{c} Precision=\frac{TP}{TP+FP} \end{array}$$ (17)

Recall measure is measured as the proportion of correct predictions generated by the model in comparison to the overall number of true occurrences:

$$\begin{array}{c} Recall=\frac{TP}{TP+FN} \end{array}$$ (18)

The F1 score measure can be computed by taking the harmonic mean of recall and precision:

$$\begin{array}{c} F1=2*\frac{Precision*Recall}{Precision+Recal} \end{array}$$ (19)

Additional factors, including the duration of training and testing, the layer and node count, and the epoch number, are assessed to facilitate a comparative evaluation between the suggested EB approach and conventional ANN techniques.

### Results with balanced data

During our initial experiments, we found that the dataset was slightly imbalanced. To address this issue, we used the SMOTE to balance the data, and the results are described in this section. The performance of the applied machine learning models with the balanced dataset is illustrated in Table 5. The analysis shows that the highest accuracy score achieved with the balanced dataset is 89% using the Random Forest (RF) method. However, these seemingly high scores may indicate low performance, possibly due to the introduction of noise from the newly generated synthetic dataset.

**Table 5:** The testing results of applied neural network methods for fault detection with balanced dataset.

Method	Accuracy	Target Class	Precision	Recall	F1
RF	0.89	No Fault (0)	0.82	1.00	0.90
		Fault (1)	1.00	0.79	0.88
		Average	0.91	0.89	0.89
LR	0.63	No Fault (0)	0.61	0.70	0.65
		Fault (1)	0.65	0.56	0.60
		Average	0.63	0.63	0.63
SVC	0.68	No Fault (0)	0.60	1.00	0.75
		Fault (1)	1.00	0.36	0.53
		Average	0.80	0.68	0.64

### Results with original data

The performance results of the applied learning models with the original dataset features are analyzed in this section. In previous analyses, we concluded that the synthetic generation of balanced datasets achieved moderate performance scores. The results with the original dataset features are demonstrated in Table 6. This analysis reveals that the applied learning models achieved good performance scores. The applied tree-based RF model achieved a notable performance score of above 90%. The applied linear models, LR and SVC, achieved lower performance scores. The neural network-based GRU achieved a high-performance score of 92% in this analysis. This analysis infers that the applied machine learning models achieve good performance scores using the original dataset features for electrical fault detection in transmission systems.

**Table 6:** The testing results of applied neural network approaches for fault detection.

Method	Accuracy	Target Class	Precision	Recall	F1
RF	0.90	No Fault (0)	0.85	1.00	0.92
		Fault (1)	1.00	0.78	0.88
		Average	0.92	0.90	0.90
LR	0.74	No Fault (0)	0.68	1.00	0.81
		Fault (1)	1.00	0.42	0.59
		Average	0.84	0.71	0.70
SVC	0.63	No Fault (0)	0.63	0.81	0.71
		Fault (1)	0.64	0.42	0.51
		Average	0.64	0.63	0.62
GRU	0.92	No Fault (0)	0.87	0.99	0.93
		Fault (1)	0.99	0.82	0.90
		Average	0.93	0.92	0.92

**Fig 7:**
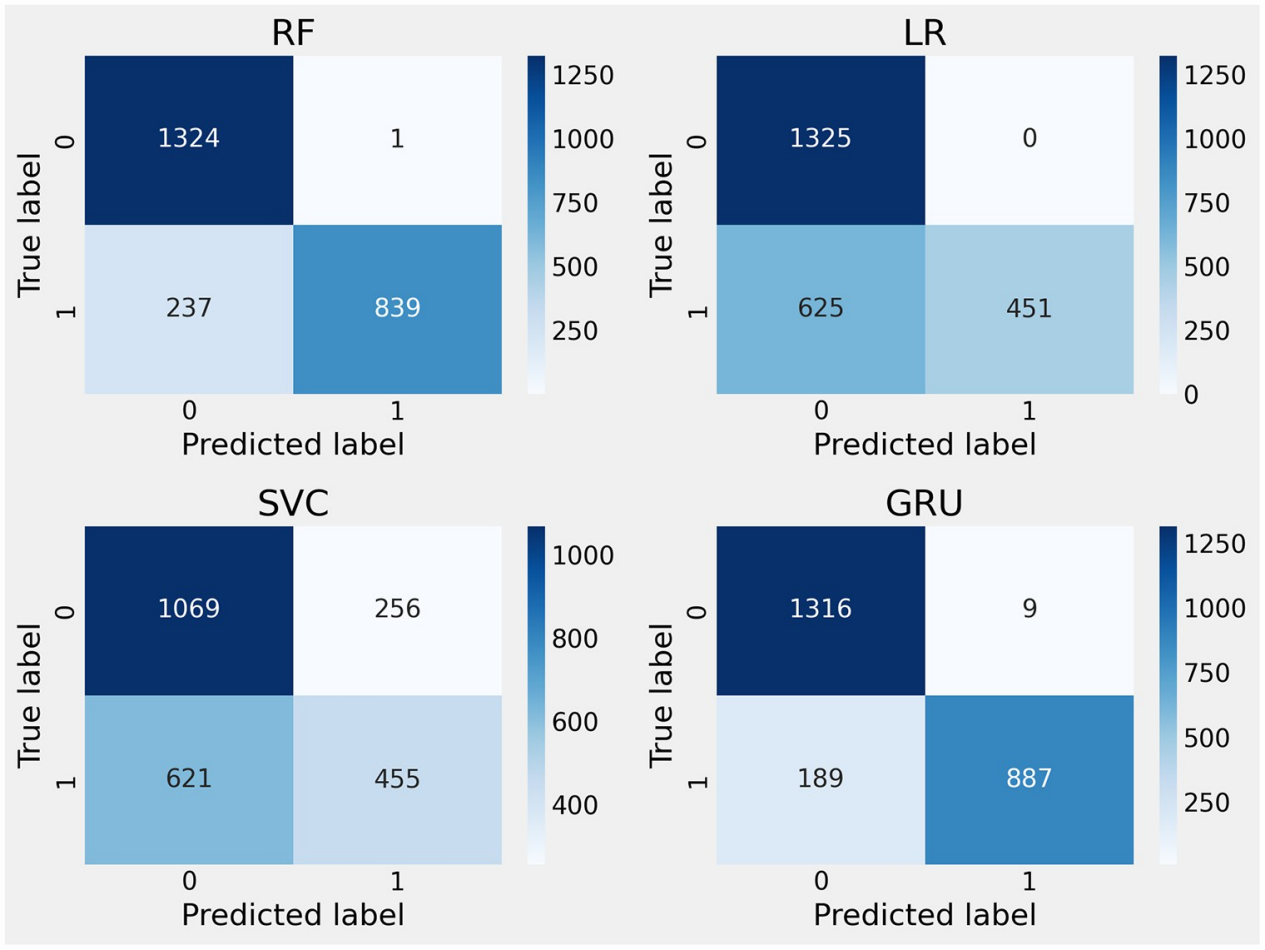
The confusion matrix-based error rates analysis for fault detection.

The confusion matrix-based performance assessment of applied neural network models for electrical fault classification is illustrated in Fig 7. The analysis reveals that during the testing phase, the applied machine learning models, LR and SVC, recorded the highest rates of incorrect predictions. In contrast, the RF and GRU models achieved lower rates of incorrect predictions. Thus, this analysis concludes that tree-based and neural network models demonstrate superior performance scores for fault detection.

### Performance analysis of proposed method

The performance results of the proposed EB approach are analyzed in this section. The results of the EB approach are presented in Table 7. The analysis expresses that our optimized EB approach achieved 99% accuracy performance scores compared to other applied methods. The class-wise performance results show the exceptional performance of the EB approach. This analysis infers that our novel proposed method outperformed other applied methods with high electrical fault detection rates.

**Table 7:** The testing results of proposed EB method for fault detection.

Proposed	Accuracy	Target Class	Precision	Recall	F1
EB	0.99	No Fault (0)	0.99	1.00	1.00
		Fault (1)	1.00	0.99	0.99
		Average	1.00	0.99	0.99

**Fig 8:**
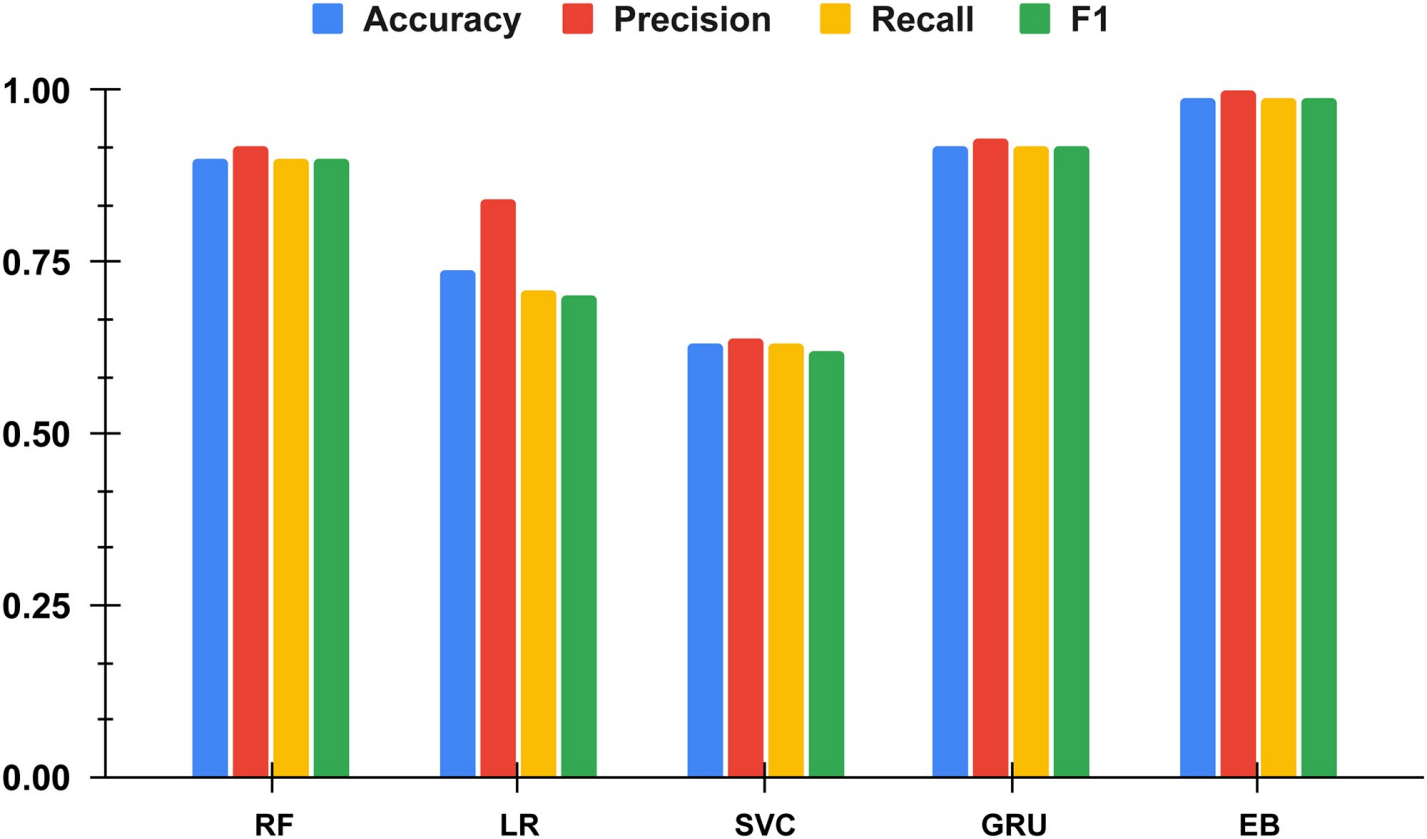
The histogram-based results comparisons of applied machine learning models.

The comparison of histogram-based results of applied machine learning models was conducted to assess the overall performance relative to the proposed approach. Fig 8 illustrates the histogram comparison results, indicating that the machine-learning-based models LR, SVC, and RF achieved lower performance results. However, the deep learning-based neural network model, GRU, achieved respectable scores but not as high as our proposed method, EB. This analysis highlights the significant performance advantage of our proposed approach in electrical fault classification.

**Fig 9:**
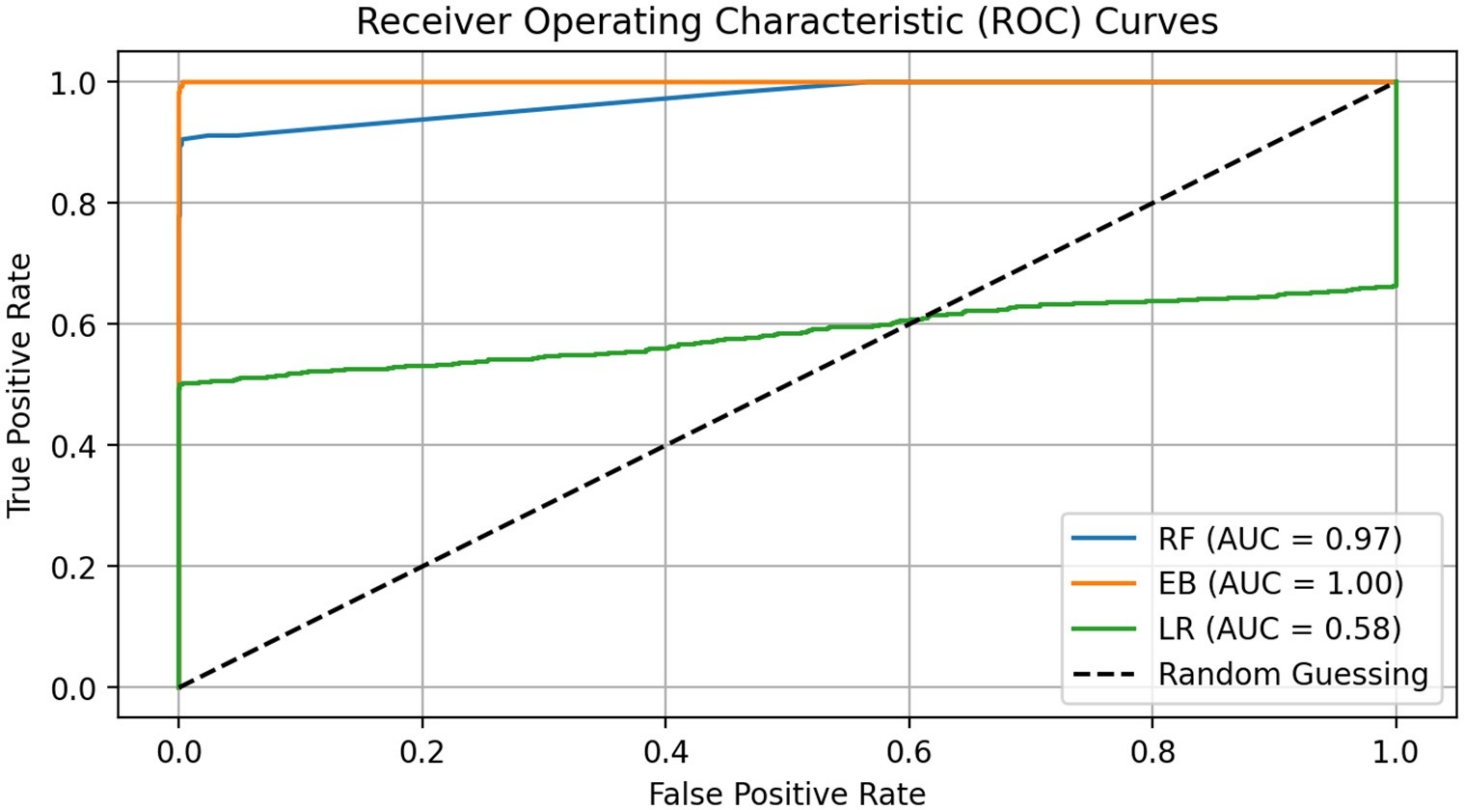
The ROC curve analysis of applied neural network methods.

Fig 9 demonstrates the assessment of the performance of applied neural network models through Receiver Operating Characteristic (ROC) analysis. The analysis reveals distinct variations in the Area Under Curve (AUC) scores, highlighting the effectiveness of the different models in accurately classifying electrical faults. The LR model exhibited a modest AUC of 0.58, indicating its relatively weak capability in effectively distinguishing between the classes. In contrast, the RF model marked a significant improvement, achieving an AUC of 0.97, which suggests a high degree of accuracy in fault classification. Most notably, our proposed model, referred to as EB, surpassed the conventional models by securing an AUC of 0.99. This superior performance of the EB model, as depicted in the ROC plot, illustrates its exceptional ability to correctly classify the positive class from the negative, establishing it as a highly reliable model for electrical fault classification. Thus, the ROC plot analysis provides compelling evidence of the advanced discriminative power of the EB model over traditional machine learning approaches.

**Fig 10:**
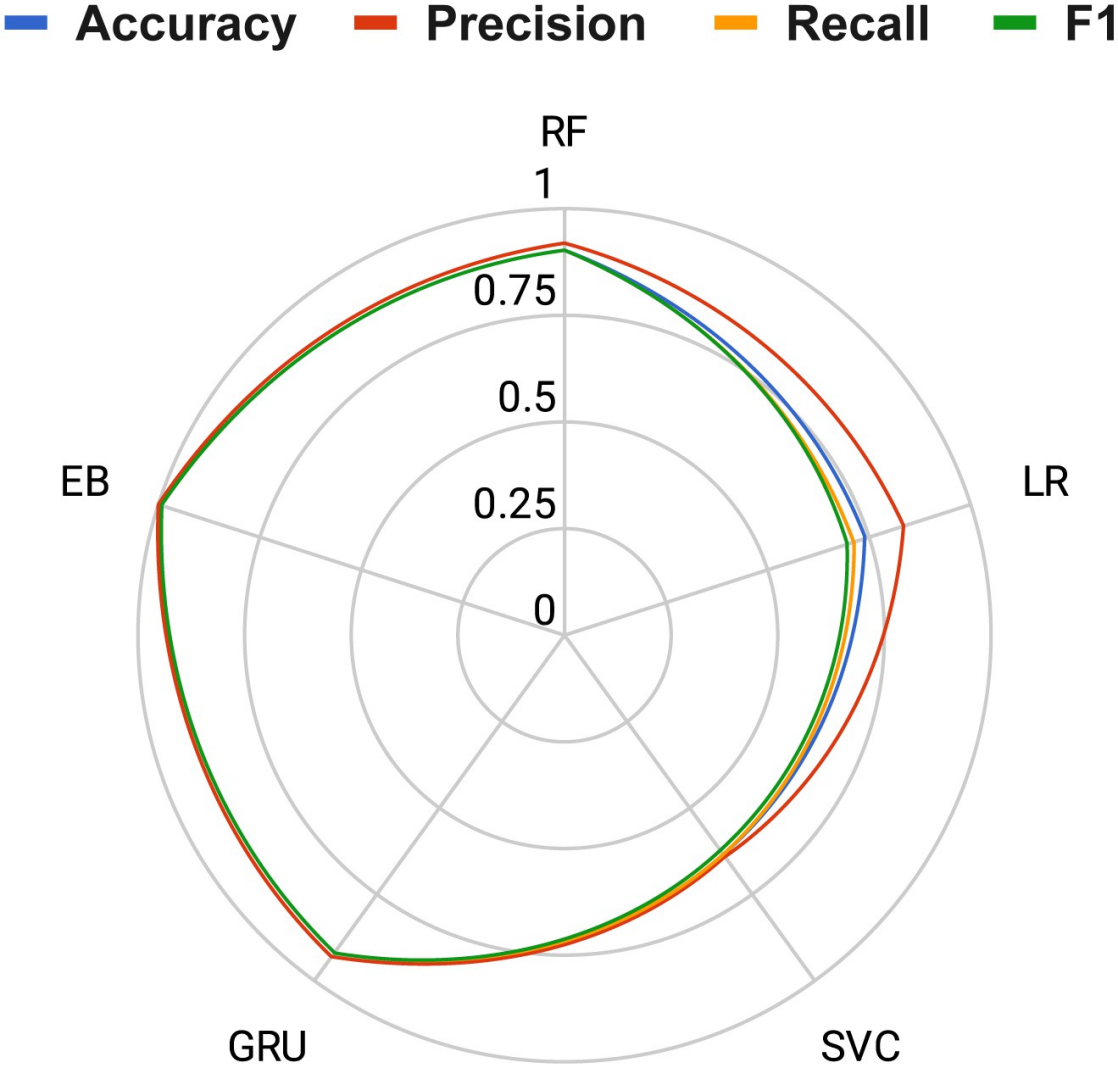
The radar chart-based performance area coverage analysis.

In addition, we have provided a radar chart-based performance area analysis, as shown in Fig 10. This analysis, based on performance metrics coverage across the radar, demonstrates that our proposed approach covers more performance scores on the radar compared to other applied methods. It also highlights the superior performance results scores of the proposed EB method for electrical fault classification.

**Fig 11:**
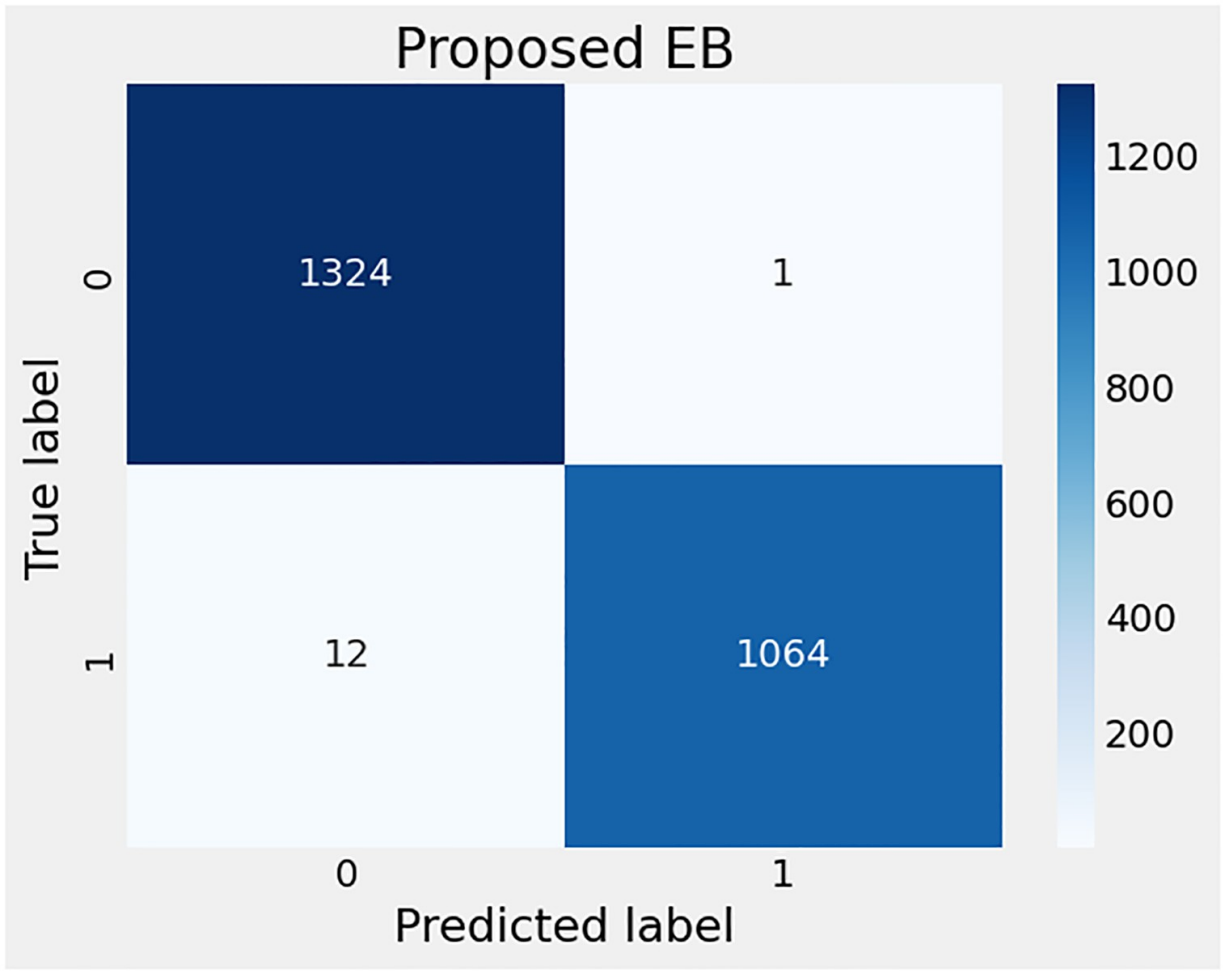
The confusion matrix-based error rates analysis of proposed EB method for fault detection.

In evaluating the performance results of the proposed EB model, the confusion matrix is illustrated in Fig 11. Remarkably, the plot reveals that the model has achieved an impressive level of precision, with only 13 instances of misclassification. This outcome underscores the model’s robustness and effectiveness in accurately distinguishing between categories, affirming its potential for widespread application in scenarios where precision is paramount. The low number of misclassifications is indicative of the model’s advanced learning capabilities and its suitability for handling complex classification challenges, such as electrical fault classification in transmission systems.

### K fold cross-validation analysis

We employed the K-fold validation technique to authenticate the performance scores of the applied method. In this method, the fault-related signal data are divided into k equal-sized folds, and the method is trained and validated k times, with each fold being used once as a validation set. This approach helps evaluate the model’s performance and addresses issues such as overfitting. The results of the K-fold approach for applied neural network methods are reported in Table 8. The analysis shows that the applied method achieved lower K-fold accuracy scores than our proposed EB approach. This analysis validates the high-performance scores of our proposed EB method, which achieved 99% K-fold accuracy with a minimum standard deviation score of 0.0009 for electrical fault detection.

**Table 8:** The k-fold-based performance validations of applied machine learning methods for fault detection.

Method	K-fold Accuracy	Standard Deviation (+/-)
RF	0.89	0.0086
LR	0.72	0.0137
SVC	0.70	0.0291
**EB**	**0.99**	**0.0009**

### Fault detection time computational complexity analysis

The computational complexity analysis of fault detection time for applied artificial neural networks is presented in Table 9. We measured the training time of each applied method and reported the results. The analysis shows that RF achieved the minimum electrical fault detection time; however, the performance accuracy of RF is low. The fault detection time of the proposed EB model is 23.5848 seconds, which is high compared to others; however, the accuracy score of the proposed approach is also 99%. We consider performance score a priority for this critical electrical fault detection. We will further reduce the architecture of the proposed EB to reduce its computational time.

**Table 9:** The fault detection training time analysis of applied methods.

Applied Method	Fault Detection Training Time
RF	0.10008 Seconds
LR	0.2102 Seconds
SVC	0.1887 Seconds
GRU	12.2944 Seconds
EB	23.5848 Seconds

### eXplainable Artificial Intelligence (XAI)

**Fig 12:**
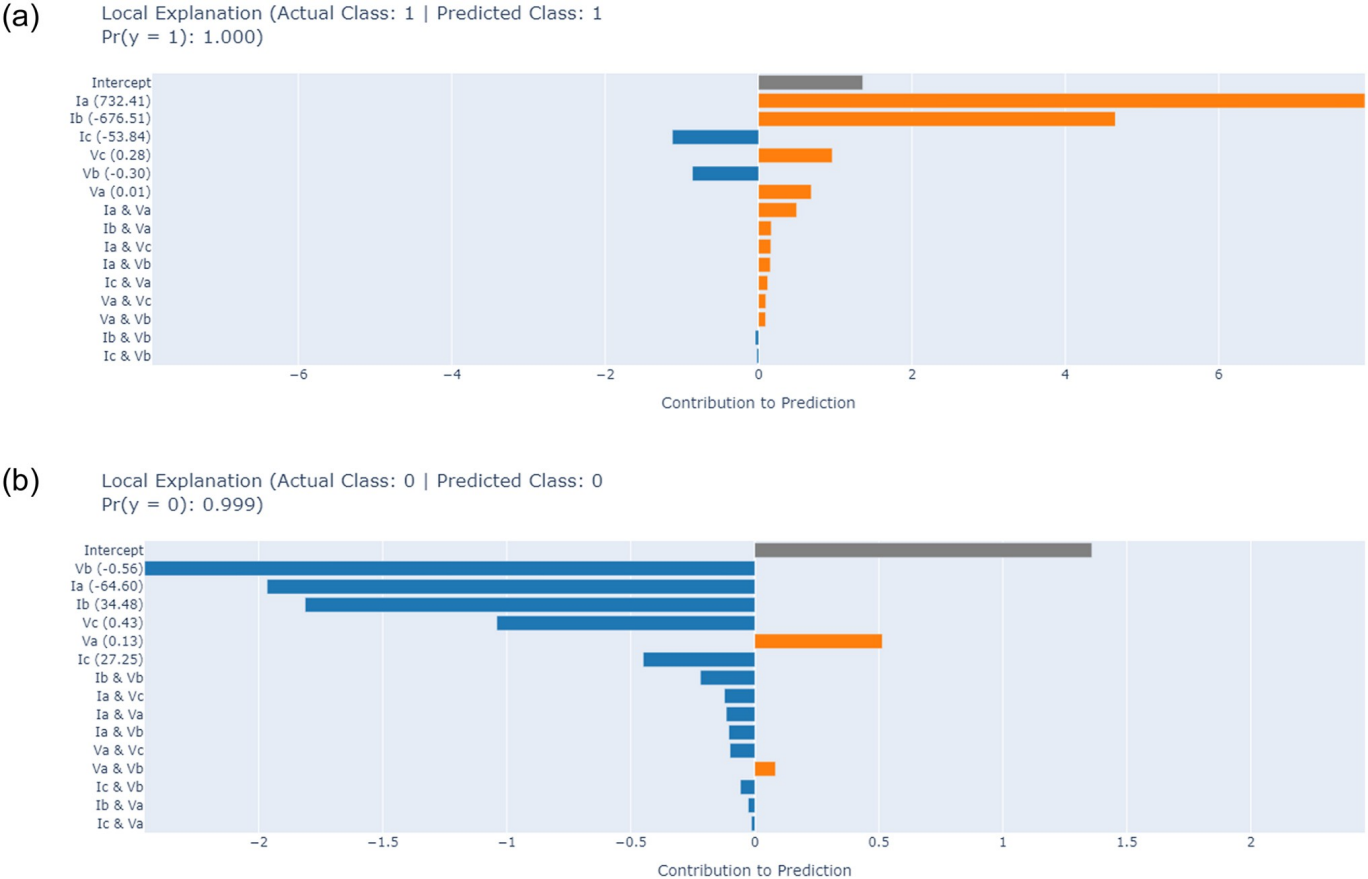
The SHAPE chart-based XAI analysis of proposed EB method for fault detection.

The implementation of the XAI approach [30] in our study has yielded significant advancements in understanding and interpreting the decision-making processes of the proposed model used for fault detection in electrical transmission systems. Through the application of the XAI technique, as shown in Fig 12. We can demystify the operational logic of the proposed model, particularly shedding light on how these models discern and classify different types of faults. This transparency not only enhances trust in AI-driven systems but also facilitates a deeper insight into the strengths and limitations of the employed models. Most notably, the XAI analysis revealed that certain features, such as the phase line current and voltage variations, play a pivotal role in the models’ fault detection capabilities.

### State of the art comparisons

To ensure a fair performance comparison of the proposed EB approach, we have collated the performance results with the state-of-the-art methods used for electrical fault detection. The results of the comparison studies using the same dataset are described in Table 10. The analysis shows that the previous highest accuracy achieved was 97%, which is considered low. This investigation demonstrates that our suggested EB methodology exceeds the performance of current leading techniques, achieving an impressive.

**Table 10:** The state of art research studies analysis for fault detection in electrical power transmission systems.

Ref.	Proposed Technique	Performance Accuracy
[11]	ANN Approach	78%
[12]	ANN Approach	80%
[13]	LSTM Network and SVM	97%
**Our**	**Novel EB**	**99**%

### Discussions

We have proposed an innovative research approach for electrical fault classification and detection timely:

- ANNs prove to be a reliable and efficient approach for classifying and detecting faults in electrical power system transmission lines, particularly given the growing dynamic connectivity of contemporary power transmission systems.
- Before applying an artificial neural network in a practical scenario, it is essential to thoroughly evaluate its performance, considering the specific neural network structure and learning algorithm.
- The Explainable Boosting (EB) neural networks exhibit impressive performance when trained with extensive datasets, readily accessible in power systems. Hence, the proposed method opts for EB due to its efficacy.

### Study limitations

The proposed research outperformed the state-of-the-art methods with the high performance of fault detection, however, there are still some limitations, as follows:

- The calculation time of the proposed method is higher compared to applied methods. In future, we will try to optimize the architecture of the proposed method to minimize the computational time.

## Conclusions

This paper proposed a novel glassbox-based explainable method for detecting and classifying faults within a three-phase transmission line system. The developed method employs a set of line currents and voltages as inputs to the neural networks, which are normalized based on their pre-fault values. The presented research results cover various faults, including line-to-ground, double-line-to-ground, line-to-line, and symmetrical three-phase faults, with separate neural networks designed for each fault type. Simulation outcomes demonstrate the effective performance of all proposed neural networks, affirming their practical implementability. Emphasis is placed on the significance of selecting the most suitable ANN configuration to optimize network performance. Key conclusions drawn from the research highlight the successful application of ANNs in fault detection and classification for diverse scenarios. The potential of ANN is expansive and offers room for further exploration. Enhancing the intelligence of fault detection and classification is possible through the development of appropriate intelligent techniques. This can be realized with advanced computers capable of efficiently handling substantial datasets and performing calculations swiftly.
